# Computational Insights into Flavonoids for ADAMTS-5 Exosite Inhibition in Knee Osteoarthritis: Docking, MD Simulations, and Machine Learning-Guided Structure Prediction

**DOI:** 10.3390/molecules31061016

**Published:** 2026-03-18

**Authors:** Mayurakkhi Bhatia, Nithyadevi Duraisamy, Mohammed Cherkaoui

**Affiliations:** Department of Digital Engineering and Artificial Intelligence, College of Science, Long Island University, Brooklyn, NY 11201, USA; nithyadevi.duraisamy@liu.edu (N.D.); mohammed.cherkaoui@liu.edu (M.C.)

**Keywords:** knee osteoarthritis, flavonoids, metalloproteinase inhibitors, molecular docking, MD simulation

## Abstract

The limited selectivity of most catalytic-site ADAMTS-5 inhibitors and the necessity to preserve aggrecan integrity in early-grade knee osteoarthritis require the development of selective aggrecanase inhibitors. The present study conducted rational in silico screening of flavonoids as potential ADAMTS-5 inhibitors by integrating high-throughput virtual screening, molecular docking, and molecular dynamic simulations targeting the exosite domains of ADAMTS-5 (the Disintegrin-like and spacer domain). The objective was to identify plant-derived flavonoids with favorable drug-like properties and specific interactions towards the ADAMTS-5 exosite as a more targeted alternative to catalytic-site inhibition. In this study, 847 flavonoids were screened using drug-likeness and ADME criteria to identify promising leads. The top 16 selected flavonoids were further subjected to molecular docking and SAR analysis. Of these compounds, Homoeriodictyol satisfied key drug-likeness criteria and exhibited the highest binding affinity to the Disintegrin-like domain, with a binding energy of −23.1 kcal/mol and favorable interactions. Molecular dynamics simulations of the Homoeriodictyol–ADAMTS-5 complex over 100 ns demonstrated stable binding throughout the trajectory. DCCM analysis and PCA further supported the proposed exosite-mediated modulation. To extend exosite mapping beyond the Disintegrin-like domain, this study also examined the spacer domain using a machine-learning-predicted structural model and identified key residues that contribute to ligand binding.

## 1. Introduction

Osteoarthritis (OA), a chronic and degenerative joint disease [1,2], is increasing globally as the aging population grows [3]. For a long time, osteoarthritis was considered a disorder of aging cartilage; it is now also recognized as a complex, multifactorial condition [4] involving mechanical stress, inflammatory mediators, and structural degradation of joint tissues [5,6]. Today, OA affects more than 600 million individuals worldwide, making it a major public health concern [7]. Knee osteoarthritis is the most common form, accounting for nearly 300 million OA cases and affecting all individuals, especially women aged 45 and older [8]. Although extensive research has been conducted over several decades, most existing treatments focus on symptom management rather than preventing cartilage degradation. There is an urgent need for targeted therapies, as most of the disease-modifying osteoarthritis medications (DMOADs) continue to represent an unmet clinical need [9].

A defining feature of osteoarthritis (OA) is the deterioration of articular cartilage integrity. Articular cartilage consists mainly of type II collagen and aggrecan, which bestow strength, elasticity, and load-bearing capacity [10]. Early cartilage deterioration and disease progression are caused by the loss of aggrecan from the extracellular matrix, largely mediated by aggrecanase- and zinc-dependent proteases belonging to the ADAMTS (a Disintegrin and metalloproteinase with thrombospondin motifs) family [11]. Among these, ADAMTS-4 and ADAMTS-5 are critically involved in aggrecan cleavage, with ADAMTS-5 exhibiting particularly high proteolytic activity under inflammatory conditions [12].

Initial biochemical characterization by Tortorella et al. (2002) revealed that human ADAMTS-5 cleaves aggrecan at several specific sites, including the Glu373-Ala374 bond, and exhibits a substrate specificity that is similar yet distinct from ADAMTS-4 [13]. Glasson et al. (2005) demonstrated that ADAMTS-5 knockout mouse models confer stronger protection against cartilage degradation [14]. This shifted the focus towards ADAMTS-5 as a DMOAD target. In a 2012 study, Deng et al. designed a potent small-molecule inhibitor of ADAMTS-5 [15]. These findings collectively underscore ADAMTS-5 as a critical therapeutic target for knee osteoarthritis.

Previous efforts to inhibit ADAMTS-5 have largely focused on active-site zinc chelation. Although these catalytic site inhibitors have in vitro potency, they frequently lack selectivity due to the conserved zinc-binding domains across the ADAMTS family [15]. Recent structural studies have elucidated the multidomain organization of ADAMTS-5, which consists of a signaling peptide [16], a pro-domain, a metalloproteinase (MP) domain, a Disintegrin-like (Dis) domain, the thrombospondin type 1 repeat (TRS), a cysteine-enriched region, a spacer (Sp) domain, and a second TRS motif [17]. Consequently, owing to the lack of selectivity at the catalytic site, exosites offer a promising approach to develop more selective and effective ADAMTS-5 inhibitors [18,19].

Recent studies by Santamaria et al. (2021) uncovered a novel small molecule (a glucosamine-binding aryl sulfonamide, denoted as **4b**) that selectively inhibits ADAMTS-5 at the MP/Dis region of the exosite [19]. However, the spacer domain remains unexplored due to a lack of structural data [20].

In this context, naturally occurring flavonoids have emerged as attractive candidates for ADAMTS-5 modulation [21]. Flavonoids are the largest class of polyphenols, found in a wide variety of fruits and other plant-based foods [22]. Flavonoids have various beneficial properties, including anti-inflammatory and anti-cancer effects, and act as antioxidants. Numerous naturally occurring polyphenolic compounds, such as flavonoids, have been found to have aggrecanase inhibitory properties and to possess metzincin inhibitory activity [23]. To date, no studies have systematically mapped flavonoid interactions across the exosite domain of ADAMTS-5.

**Scheme 1:**
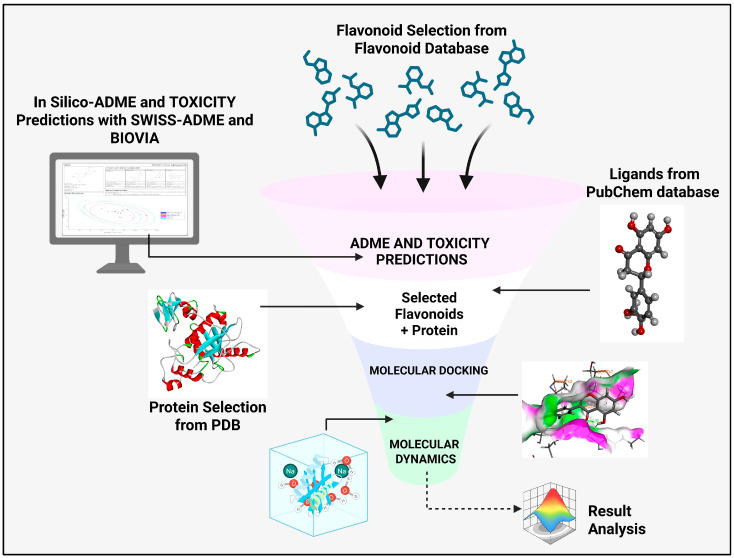
Schematic representation of the methodology utilized in this study, beginning with high-throughput virtual screening, proceeding through docking, scoring, and molecular dynamics simulation.

In this study, we identify exosites within the ADAMTS-5 Dis domain as a major pharmacological target, and, for the first time, explore the spacer (Sp) domain to elucidate the molecular interactions of flavonoids as potential ADAMTS-5 inhibitors for the treatment of knee osteoarthritis. The computational approach combines structure-based virtual screening to identify and design selective ADAMTS-5 inhibitors. Scheme 1 provides a schematic overview of the study.

## 2. Results

### 2.1. Development of a Flavonoid Screening Library

The flavonoid database yielded a list of 847 flavonoids belonging to the following subfamilies: Flavonol (170), Anthocyanin (146), Flavanone (121), Flavanol (76), Isoflavanone (188), Flavone (137), Dihydrochalcones (1), Dihydroflavonol (1), and Chalcones (7). The selected flavonoids were filtered from the NCBI website.

### 2.2. Drug-Likeness and In Silico ADMET Scoring Values

Drug-likeness and lead-likeness were evaluated by considering flavonoids that violated none of the Lipinski Rule of Five, Ghose, Veber, Egan, and Muegge filters, and that triggered no PAINS alerts. The filtered flavonoids were selected for further in silico ADME and toxicity studies. ADMET_Solubility_Level scores of 3 and 4 indicated good and optimal drug-likeness, respectively. ADMET_BBB scores of 2, 3, and 4 indicated medium, low, and undefined levels of penetration, respectively. A TOPKAT_Ames_Mutagenicity probability near 1 indicated a high probability of mutagenicity, while a value near 0 indicated a low probability.

**Figure 1:**
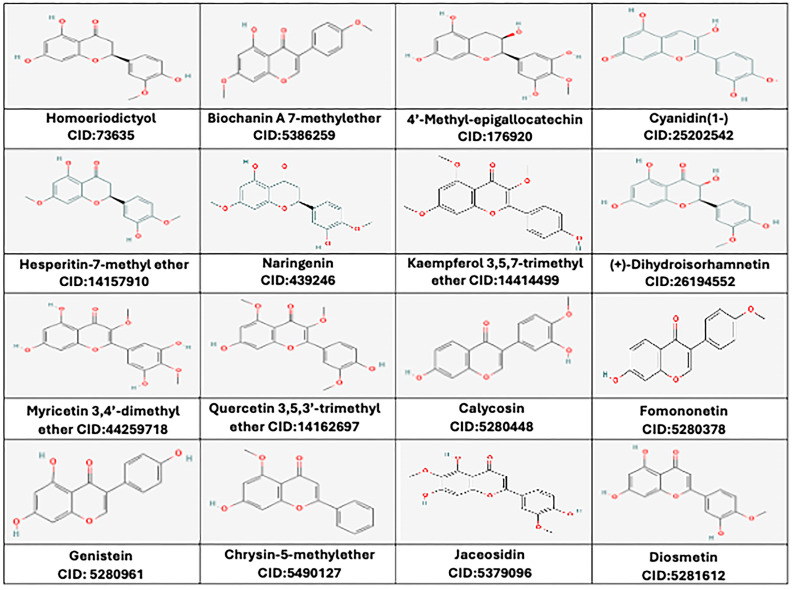
Two-dimensional structural representation of 16 flavonoids obtained from the Pubchem database selected through virtual screening, shown along with their chemical identification numbers.

The ADME and toxicity studies led to the selection of 16 potential flavonoids: 4′-methyl Epigallocatechin, Cyanidin (1-), Homoeriodictyol, Hesperetin-7-methyl-ether, Naringenin, Kaempferol 3,5,7-trimethyl ether, (+)-Dihydroisorhamnetin, Myricetin 3,4′-dimethyl ether, Quercetin 3,5,3′-trimethyl ether, Biochanin A 7-methyether, Calycosin, Fomononetin, Genistein, Chrysin-5-methylether, Jaceosidin, and Diosmetin. The compound IDs of all the ligands and their 2D structures are illustrated in Figure 1. The physicochemical properties and in silico ADMET scores of these 16 flavonoids are given in Supplementary Tables S1 and S2. Finally, the 3D structures of these 16 potential flavonoids were obtained from PubChem and subjected to in situ ligand minimization before molecular docking.

### 2.3. Defining Binding Sites at the Dis Domain

The structure of ADAMTS-5 was analyzed using PDB entry 2RJQ, which contains the amino acid sequence from SER264 to ASP555 in the A chain, with a resolution of 2.60 Å, and co-crystallized with the inhibitor Batimastat. The protein structure was prepared by removing all other ligand molecules. In the A chain, amino acid positions from 485 to 566 correspond to the Dis Domain of ADAMTS-5.

### 2.4. Molecular Docking Analysis, Binding Affinity Scores, and SAR Analysis at the Dis Domain

**Figure 2:**
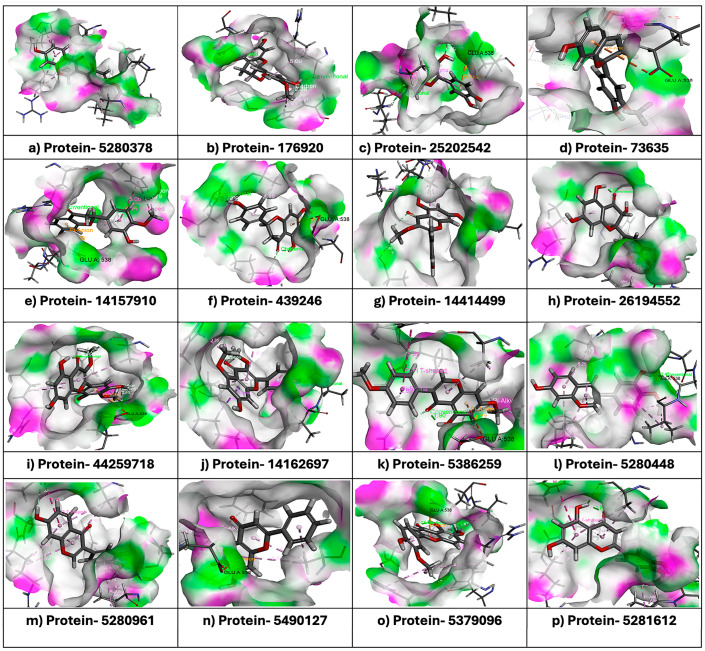
Prediction of the binding interaction site between 16 flavonoids (**a**–**p**) and the ADAMTS-5 protein complex through molecular docking analysis utilizing Biovia Discovery Studio (v24.1.0.321712) from Dassault Systèmes.

**Figure 3:**
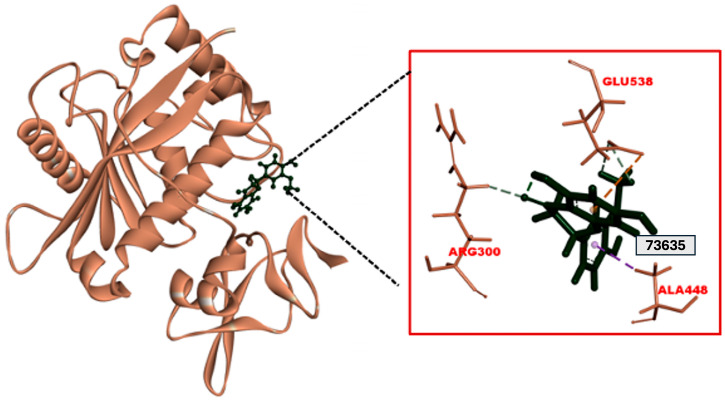
Representation of the molecular interaction of Homoeriodictyol (PubChemID: 73635) within the binding site of the ADAMTS-5 structure, interacting with the GLU538, ARG300, and ALA448 residues.

A molecular docking study was performed to assess interactions between Disintegrin-like (Dis) domains and the selected set of 16 flavonoids, the reference catalytic site inhibitor BAT (Batimastat), and the reference Exosite inhibitor Arylsulfonamide (**4b**). The docking study was performed using the CDocker algorithm; the lower the CDocker energy, the better the ligand–protein binding, as shown in Table 1. The binding pocket of the Dis domain was depicted as containing GLU538, one of the key residues for interactions with most flavonoids, as shown in Figure 2a–p. Negative CDocker interaction energies indicated stable binding, as shown in Table 1. The results of molecular docking indicated that among the 16 flavonoids, Homoeriodictyol demonstrated the highest CDocker energy (−23.10 kcal/mol) and a CDocker interaction energy of −29.06 kcal/mol, forming a Pi–anion interaction with residue GLU538 and a carbon–hydrogen bond with residue ARG300, as shown in Figure 3. The results also indicated that Batimastat and Arylsulfonamide had binding affinities of −28.32 kcal/mol and −10.08 kcal/mol, and CDocker interaction energies of −26.08 kcal/mol and −47.77 kcal/mol, respectively. Structure–activity relationship (SAR) analysis was performed for the selected ligands based on the structural integrity. The analysis suggested that the number of methoxy and hydroxyl groups on the flavonoids played a distinct role in the inhibition. Hydroxyl groups (-OH) formed hydrogen bonds with residues such as GLU538, ARG300, and HIS304. Flavonoids with the most hydroxyl groups, like 4′-Methyl-Epigallocatechin, Homoeriodictyol, Cyanidin (1-), and Kaempferol 3,5,7-trimethyl ether, formed hydrogen bonds that stabilized the binding of the ligand. Methoxy (-OCH_3_) groups in flavonoids such as Homoeriodictyol, Biochanin A 7-methyl ether, and Kaempferol 3,5,7-trimethyl ether contributed to hydrophobic interactions with residues such as PHE406 and PRO535. Compounds with both hydroxyl and methoxy groups demonstrated a balanced interaction profile, as shown in Table 1. Therefore, based on the favorable binding energy value and its interactions with key exosite residues, Homoeriodictyol was selected as a candidate for molecular dynamics simulation.

### 2.5. Molecular Dynamics Simulation Results at the Dis Domain

**Figure 4:**
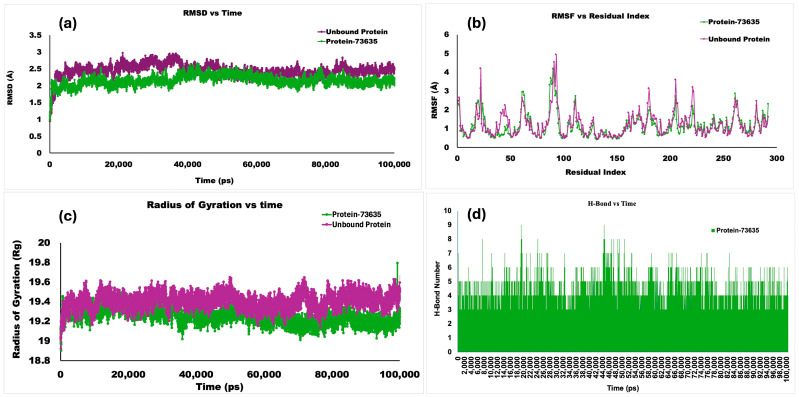
Molecular dynamics simulation trajectory analysis of Homoeriodictyol (PubChemID: 73635), in complex with the ADAMTS-5 protein over a 100 ns simulation period: (**a**) Root Mean Square Deviation (RMSD) profiles illustrating structural stability and conformational changes in the Homoeriodictyol–ADAMTS-5 complex in comparison to the unbound protein, (**b**) plots of Root Mean Square Fluctuation (RMSF) demonstrate residue-specific flexibility for both the unbound and protein–ligand complex, (**c**) the Homoeriodictyol-bound ADAMTS-5 complex’s structural rigidity and folding activity when compared to the unbound protein during the 100 ns MD simulation are represented by the Radius of Gyration (Rg) analysis, (**d**) Homoeriodictyol and the ADAMTS-5 protein formed a number of hydrogen bonds throughout the 100 ns simulation, indicating the stability of the interaction inside the binding surface.

Molecular dynamics (MD) simulations were performed in BIOVIA Discovery Studio for the Dis domain of ADAMTS-5, both in the absence of ligand (unbound protein) and as a protein–ligand complex with Homoeriodictyol, on a 100 ns timescale. The protein’s RMSD progression is displayed in Root Mean Square Deviation (RMSD) graphs. The reference frame’s c-alpha backbone frame was used as the first alignment point for all protein frames. The RMSD values, which were within a 3 Å threshold (Figure 4a), revealed that all complexes—both the unbound protein and the protein docked with the ligand—remained very stable, since lower RMSD values imply greater stability of the complex.

The results demonstrated the stability of both the unbound protein and the ligand-bound complex for the 100 ns simulation period, with average RMSDs of 2.44 Å and 2.14 Å, respectively. Root Mean Square Fluctuation (RMSF) analysis was conducted, as shown in Figure 4b. RMSF was used to re-evaluate and understand the fluctuations in the ligand-bound protein complex structure. The unbound protein ADAMTS-5 showed the greatest fluctuation of 4.92 Å at GLU356. The protein–ligand complex showed the highest fluctuation of 4.17 Å, with the highest peaks at ASP353. Most residues in the protein–ligand complex were conserved compared to the unbound protein.

The Radius of Gyration (Rg) values for the protein–Homoeriodictyol complex and the unbound protein demonstrated the compactness of the complex (Figure 4c). During the dynamic simulation, intermolecular hydrogen-bonding interactions between the ligand and important protein residues were assessed to determine the complex’s strength. The results showed that more than two hydrogen bonds formed, and even up to nine formed, within a 100 ns time frame (Figure 4d).

**Figure 5:**
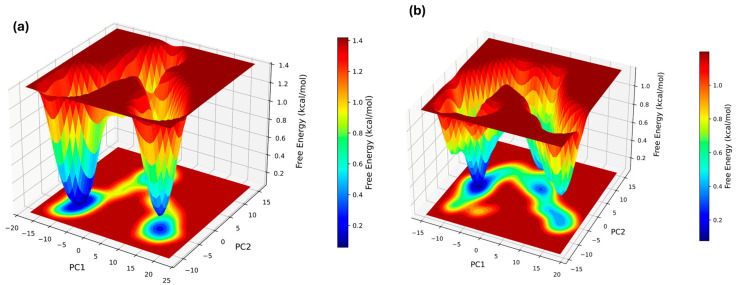
(**a**) Free-energy landscape (FEL) plots derived from PCA for the Homoeriodictyol-bound ADAMTS-5 complex based on 100 ns MD simulations, (**b**) free-energy landscape (FEL) plots derived from PCA of the unbound protein.

The Principal Component Analysis (PCA) of the free-energy landscape projections (FEL plots) revealed that the ligand-bound complex occupies a restricted conformational space, as indicated by a deeper, narrower energy well (Figure 5a) compared to the unbound protein with a broader energy landscape (Figure 5b). The results revealed that Homoeriodictyol interacted with a few stable protein conformational states.

**Figure 6:**
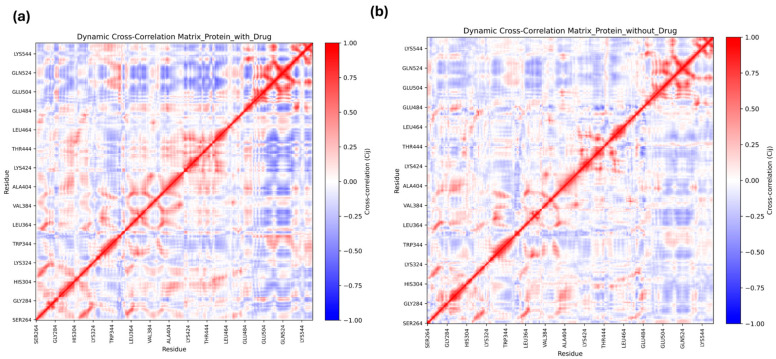
(**a**) Dynamic Cross-Correlation Matrix (DCCM) of ADAMTS-5 bound with Homoeriodictyol from a 100 ns MD trajectory, (**b**) Dynamic Cross-Correlation Matrix (DCCM) of the unbound protein.

Post-MD Dynamic Cross-Correlation Matrix (DCCM) analysis was performed. The results highlighted the differences in the correlated motions between the ligand-bound complex and the unbound protein. The Homoeriodictyol-bound complex revealed more correlated regions (indicated by red clusters) than the unbound protein, with diffuse patterns (Figure 6a vs. Figure 6b). These results highlighted that exosite interaction with flavonoids may influence the protein’s catalytic behavior.

### 2.6. Calculations of Ligand–Protein Binding Energy (MM-GBSA)

The MM-GBSA calculated for the ligand–protein complex before and after a 100 ns simulation was −43.02 kcal/mol and −42.61 kcal/mol, respectively. The residue interaction analysis conducted after a 100 ns simulation supported that residues such as ALA448, ASP447, ALA305, GLU538, HIS304, ARG300, SER449, and GLN550 contributed to H-bond occupancy throughout the 100 ns simulation, as shown in Supplementary File Figure S1. The results highlighted that around 41% of H-bond occupancy was due to the residue ALA448, 6% to ASP447, and 1% to GLU538.

### 2.7. Findings in the Spacer Domain

**Figure 7:**
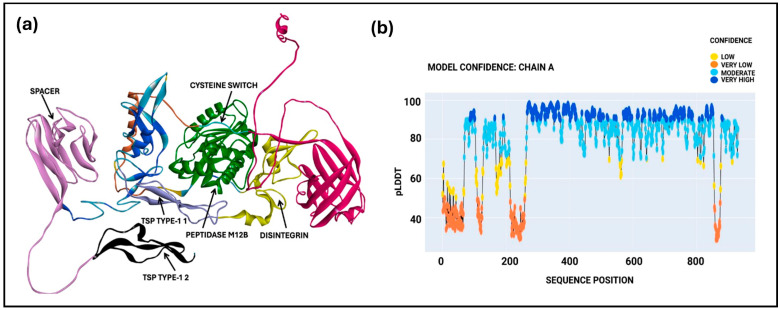
(**a**) The predicted protein structure of ADAMTS-5 with the spacer domain, (**b**) the model confidence metric for the predicted protein structure.

The spacer domain was explored using a machine-learning-driven OpenFlow algorithm, using the FASTA sequence (Uniprot ID: Q9UNA0) from the Uniprot database to predict the ADAMTS-5 Sp domain structure. The modeler program predicted a single model with a mean pLDDT score of 78.79, which is within the moderate confidence range. The Predicted Local Distance Difference Test (pLDDT) provides a confidence score for protein structure prediction using machine learning tools. The predicted structures, including all domains (Figure 7a) and the model confidence metric, including the pLDDT score from OpenFold, are shown in Figure 7b.

Further evaluation with the Verify-3D model tool yielded a score of 365.13, which is close to the expected high value and suggests a high-quality protein model. The Sp domain of ADAMTS-5 (residues 732–874) was visualized in BIOVIA Discovery Studio and utilized for ligand interaction studies. A Ramachandran plot of the model showed that most of the spacer domain regions fall in the β-sheet region (highlighted yellow dots), as shown in the Supplementary File (Figure S2). The structural mapping of the predicted spacer domain containing residue SER824 is within the hypervariable loop. To further understand potential interactions within the Sp domain (amino acid sequence 734–855), the 16 flavonoids were docked. Among these, (+)-Dihydroisorhamnetin formed a hydrogen bond with SER824 in the Sp domain, exhibiting the best CDocker energy of −15.21 kcal/mol and CDocker interaction energy of −26.64 kcal/mol, as detailed in Supplementary Table S3. The interaction of the flavonoid with SER824 suggests that ligand binding at this site may have an impact on substrate recognition. The results from the short 10 ns MD simulation indicated greater flexibility, necessitating further refinement in future studies. The RMSD and RMSF results from the 10 ns MD simulation are shown in the Supplementary File (Figure S3). These results also indicate the possibility of targeting the Sp domain for further application in the treatment of knee osteoarthritis.

## 3. Discussion

ADAMTS-5 is important in the development of early-stage osteoarthritis (OA) as a major enzyme responsible for cartilage breakdown through aggrecan degradation [24]. It is crucial to pursue novel therapeutic approaches, as most ADAMTS-5 inhibitors developed to date have shown limited selectivity and poor clinical efficacy [23]. Aggrecanase inhibition can be mediated through two main mechanisms: exosite binding at the enzyme’s distal regions or zinc chelation at the catalytic site [17]. The selectivity of zinc chelators is limited by the conserved nature of ADAMTS-5 family members, whereas exosite-targeting small molecules, as demonstrated by Santamaria et al., achieve better specificity through interaction with the unique substrate-binding interface formed by the Disintegrin-like and catalytic domains [18].

Flavonoids have been recognized as promising candidates for aggrecanase inhibition due to their dual metzincin-inhibitory and anti-inflammatory characteristics. Aggrecanase activity is suppressed by luteolin in in vitro and in vivo studies [20,23]. Intrigued by these findings, this work utilizes virtual screening-based computational analysis to examine flavonoids as potential ADAMTS-5 exosite inhibitors.

To speed up lead identification, computational methods such as molecular docking and molecular dynamics simulations are increasingly used in modern drug development [25]. Complementing previous work on small-molecule-targeting exosite therapies, this study was designed to extend the skeleton to natural flavonoids with potential inhibitory activity against the ADAMTS-5 exosite. Screening 847 flavonoids for physicochemical and toxicity parameters identified 16 candidates that satisfied Lipinski’s Rule of Five, indicated acceptable drug-likeness, and were predicted to be non-mutagenic.

This study focused on the Dis domain of ADAMTS-5, which is important for its enzymatic activity. Molecular docking of 16 flavonoids and the reference ADAMTS-5 inhibitor with the Dis domain showed that Homoeriodictyol had the highest CDocker energy of −23.10 kcal/mol and formed a key interaction with GLU538, ARG300, and ALA448. The binding affinity of Homoeriodictyol was compared with two reference inhibitors: a small-molecule ADAMTS-5 catalytic site inhibitor (BAT) and an exosite inhibitor, Arylsulfonamide (**4b**). BAT is a catalytic site inhibitor, and hence, although exhibiting higher CDocker energy, it has limited selectivity. In contrast, although Homoeriodictyol demonstrated slightly lower CDocker energy, its interaction at the Dis domain is advantageous as it can reduce off-target effects. The binding affinity of Homoeriodictyol was also compared with Arylsulfonamide (**4b**), and the CDocker energy was −10.08 kcal/mol, which was lower than that of Homoeriodictyol. This indicates the potential of Homoeriodictyol’s pharmacophore for future medication design for knee osteoarthritis.

Structure–activity relationship (SAR) analysis of the screened flavonoids indicated the significance of the distribution of hydroxyl and methoxy groups in the ortho and para positions in influencing ligand interaction within the ADAMTS-5 Dis domain. The hydroxyl groups contribute to hydrogen bonding and help stabilize the ligand with polar residues. Methoxy substitutions contribute to hydrophobic interactions with the exosite and with non-polar residues [26]. Flavonoids containing both hydroxyl and methoxy groups, like Homoeriodictyol (one OCH_3_ and three OH groups), exhibit a balanced interaction profile, indicating their importance for ligand binding. The binding of flavonoids via both hydrogen and hydrophobic interactions may also result create steric hindrance, preventing substrate recognition at the catalytic site. Furthermore, molecular docking is a static model and does not account for protein flexibility and dynamics. Therefore, based on the desirable binding energy value and interaction profile, Homoeriodictyol was considered as a candidate for molecular dynamics simulation.

Molecular dynamics simulations show minimal conformational changes when Homoeriodictyol binds to the Dis domain. During the 100 ns simulation, the RMSD of both the unbound and bound forms remained below 3 Å, indicating that the complex remained stable. In the same way, the Radius of Gyration [27] study confirmed that the protein’s structural uniformity was maintained following ligand binding. These findings imply that Homoeriodictyol interacts with ADAMTS-5 without disrupting its overall structure, which supports targeting the Dis domain in future drug design efforts.

In DCCM analysis, the values of −1 to +1 in the DCCM plots indicated different correlations, and the +1 (red region) highlighted positive correlations, suggesting movement of residues in the same direction. The blue region corresponding to −1 highlighted a strong negative correlation between residues moving in opposite directions. Comparison of the DCCM plots for the unbound protein and the protein–ligand complex revealed patterns of correlated and anti-correlated residue motions upon binding of Homoeriodictyol, with correlations with residues from the catalytic region, indicating communication across the domains of the protein.

Free-energy landscape plots derived from two principal components (PC1 and PC2) also revealed deeper, more localized energy for the Homoeriodictyol-bound complex, indicating stabilization in particular conformational states. DCCM analysis and PCA collectively suggest that protein dynamics might have been modulated by flavonoid binding. The relative free energy bar, with darker shades of blue, corresponds to stable energy conformations; hence, the ligand-bound complex shows deeper, localized, low-energy stable conformations. Unstable energy confirmations are highlighted in darker shades of red. The unbound protein system showed a broader energy landscape.

The lack of an experimentally resolved structure for the Sp domain of ADAMTS-5 has limited insight into its functional role. Using the machine learning/OpenFold algorithm, we predicted the structure of this domain. Given that the Sp domain is also responsible for substrate binding [28], we focused on identifying key residues involved in flavonoid interactions. These key residues within the spacer region between amino acids 732 and 874, located in the hypervariable region, were studied to understand any potential interaction with flavonoids. Although the OpenFold model-derived spacer domain is not experimentally resolved and exhibits greater flexibility in short MD simulations, it provides a hypothesis-driven framework that can guide future structure-based inhibitor design.

Our results support the hypothesis of selective inhibition at the exosite, complementing the established ADAMTS-5 inhibitor strategies. This research also demonstrates the ability of high-throughput virtual screening to expedite development of natural products, such as flavonoids, as therapeutics for the treatment of knee osteoarthritis, given the huge clinical need for disease-modifying osteoarthritis medications.

## 4. Materials and Methods

In this study, we analyzed the therapeutic potential of ADAMTS-5 exosite regions using a range of computational methods, in BIOVIA Discovery Studio (v24.1.0.321712) and Python (v3.11).

### 4.1. Flavonoid Library Preparation

A list of flavonoids considered for this study was retrieved from the Phenol Explorer 3.6 database at http://phenol-explorer.eu/ [29]. The selected flavonoids were further filtered using keyword-based searches (“Pubchem”, “Push to Entrez”, “Lipinski Rule of Five”, and “Bioassay tested positive”) on the NCBI website and evaluated based on their physicochemical and biological properties to ensure they met the necessary criteria as potential therapeutic candidates.

### 4.2. Drug-Likeness and In Silico ADMET

Drug-likeness for the selected flavonoid library was evaluated using SwissADME (https://www.swissadme.ch/) [30] by applying Lipinski’s Rule of Five, a Ghose filter, a Veber filter, an Egan filter, and a Muegge filter [31]. Lipinski’s Rule of Five considers four parameters [32] to predict drug-likeness. Specifically, the compounds were evaluated to ensure that they had a molecular weight <500, a logP value <5, fewer than 5 hydrogen bond donors, and fewer than 10 hydrogen bond acceptors [33]. The Ghose filter evaluated molecular weight, molar refractivity, and lipophilicity.

The Veber filter considered the overall polar surface area and the number of rotatable bonds. The Egan filter examined lipophilicity (WLogP). The Muegge filter evaluated molecular weight, lipophilicity, total polar surface area, number of rings, number of carbon atoms, and number of rotatable bonds. The Pan-Assay Interference Compounds (PAINS) filter resulted in false-positive predictions [34]. The compounds were further subjected to absorption, distribution, metabolism, and excretion (ADME) analysis [35], along with toxicity assessment using the TOPKAT Ames Mutagenicity tool in BIOVIA Discovery Studio (v24.1.0.321712) [36].

### 4.3. Preparation of the Dis Domain

The ADAMTS-5 exosite structure with the Dis domain (PDB: 2RJQ) was retrieved from the RCSB database. Protein structure preparation was conducted using BIOVIA Discovery Studio (v24.1.0.321712). The resulting complex was energy-minimized using the conjugate gradient algorithm for up to 10,000 steps, employing the CHARMM36 force field.

### 4.4. Molecular Docking at the Dis Domain and SAR Analysis

Molecular docking was performed to understand the interactions between the flavonoids and the ADAMTS-5 exosite domain at the atomic level. The CDOCKER method and SAR analsyis was used to examine how these flavonoids interact with the target protein [37]. The structures of the screened flavonoids were downloaded and docked against the Dis domain of ADAMTS-5. For the Dis domain receptor, the grid box binding site was defined by spheres of coordinates at x = −27.2978, y = −12.1079, and z = 8.59032, with a radius of 10.4 Å. The CDOCKER protocol generated 10 ligand poses, and the pose with the most negative CDOCKER energy was selected as the best pose. This score was used to represent the strongest-bound ligand confirmation and its binding affinity. Structure–activity relationship (SAR) analysis was conducted on flavonoid structures using the interaction analysis tool in BIOVIA Discovery Studio to understand the position of the functional groups, which might be responsible for strong interaction with the target protein.

### 4.5. Molecular Dynamics Simulation at the Dis Domain

Molecular dynamics (MD) simulation, a computational method for modeling the dynamics of atoms, was applied. The target protein (ADAMTS-5 with the Disintegrin domain), referred to as the unbound protein in the study, and the best-scoring protein–ligand complex with the highest binding affinity were selected for MD simulation. The entire system was assigned the CHARMm36 force field to describe bonded and nonbonded interactions, followed by solvation and a standard dynamics cascade protocol. The protein surface was set at least 7 nm away from the edge of the orthorombic box. Sodium (Na^+^) and chloride (Cl^−^) counterions were added to neutralize the system and achieve a salt concentration of 0.145 M.

The simulation followed a standard dynamics cascade: minimization, heating, equilibration, and production. The first minimization used the Steepest Descent algorithm for up to 1000 steps with an RMS gradient of 1. The second minimization used the Adopted Basis NR algorithm for up to 2000 steps with an RMS gradient of 0.1. Results were recorded every 10 ps during the simulated heating phase, starting at 50 K and raising the temperature to 300 K. Using a time step of two femtoseconds (fs) and saving results every 10 ps, the complex was equilibrated for 500 ps. Electrostatic interactions were calculated using the Particle-Mesh-Ewald (PME) method [27]. The production step of the MD simulation was carried out for 100 ns under constant pressure and temperature (NPT), with time steps of 2 fs and saving results every 10 ps. Trajectory analysis was used to evaluate RMSD, RMSF, and Radius of Gyration (Rg) profiles.

To understand the correlated and anti-correlated motions of the protein residues during 100 ns simulations, Dynamic Cross-Correlation Matrix analysis [38] was performed. The c-alpha atom coordinate of each residue was considered, the pdb structure and trajectory were loaded into VMD, and all frames were aligned to the first frame using the protein backbone. The DCCM was then exported to Python (v3.11) for analysis and visualization. DCCM heatmaps were generated for both ligand-bound and unbound proteins.

Principal Component Analysis (PCA), being a widely used technique, was performed post-MD simulation to understand atomic displacements and major conformational changes in the protein. In the present study, PCA was performed in Python using MDAnalysis for trajectory alignment and scikit-learn for PCA. The two principal components (PC1 and PC2) were used to represent the dominant conformational space sampled during simulation and were used to construct the free-energy landscape (FEL) plots [39].

### 4.6. Molecular Mechanism-Generalized Born Surface Area (MM-GBSA) Calculations

The MM-GBSA method uses molecular mechanics force fields with a generalized Born and surface area continuum model [27]. The CHARMm force field was used in the MM-GBSA computation, and Memory Rone was used for partial charge estimation to calculate the binding free energy of the protein–ligand complex [27]. Residue interaction analysis was conducted using Python scripts implemented with MDAnalysis, taking all the trajectory frames from a 100 ns MD simulation. The equations used to calculate MM_GBSA work are as follows:

ΔG = E_complex (minimized) − [E_ligand (minimized) + E_receptor (minimized)]
(1)

### 4.7. Exploration of Interacting Residues from the Spacer Domain (Sp) of ADAMTS5

Key residue interactions in the Sp domain with flavonoids were identified using machine-learning-driven computational tools. The protein structure of ADAMTS-5, including the spacer domain, was predicted using the OpenFold workflow in BIOVIA Discovery Studio, incorporating the corresponding FASTA sequence file obtained from the Uniprot database (Uniprot ID: Q9UNA0). The quality of the predicted protein structure was assessed using the Verify-3D model, and the stereochemical quality of the structure was evaluated using the Ramachandran plot in BIOVIA Discovery Studio. For the Sp domain receptor grid box, the binding-site sphere coordinates were: x = 4.14837, y = −2.26998, and z = −44.5033, and the radius was 5.5 Å. The screened flavonoids were docked against the spacer domain. A short 10 ns molecular dynamics relaxation simulation was conducted to understand the dynamic behavior.

## 5. Conclusions

Our study highlights a structure-based computation framework to identify natural compounds, such as flavonoids, as selective inhibitors of ADAMTS-5, an enzyme linked to cartilage degradation in knee osteoarthritis. After rigorous in silico filtering based on drug-likeness, ADME, and toxicity criteria, 16 potential flavonoids were identified from a library of 847 flavonoids. Molecular docking and structure-based analysis highlights Homoeriodictyol as the most favorable ligand binding at the Dis-domain exosite pocket. A 100 ns molecular dynamics simulation confirms the stability of the Homoeriodictyol–ADAMTS-5 complex, while DCCM analysis and PCA support an exosite-mediated inhibitory mechanism. In addition, a machine-learning model allowed preliminary exploration to identify key residues in the spacer domain. Although further biochemical validation is required, these findings may guide future exosite-focused drug design. Our research will open the door to further in vitro and in vivo investigation of Homoeriodictyol’s inhibitory effects in the treatment of knee osteoarthritis.

## Figures and Tables

**Table 1:** Summary of the CDocker energy and CDocker interaction energy, and the interacting residues between 16 flavonoids and the reference inhibitors at the Dis domain of the ADAMTS-5 protein complex.

Flavonoids	CDocker Energy (kcal/mol)	CDocker Interaction Energy (kcal/mol)	SAR Analysis for Functional Groups	Interacting Residues
Homoeriodictyol CID: 73635	−23.10	−29.06	-OCH_3_(1) -OH (3)	GLU538, ARG300, ALA 448
Biochanin A 7-methyl ether CID: 5386259	−16.50	−28.97	-OCH_3_(2) -OH (1)	GLU538, PHE406, LEU301, ALA448, ARG300, HIS304
4′-Methyl-Epigallocatechin CID: 176920	−15.38	−27.22	-OCH_3_(1) -OH (5)	GLU538, ALA448, ARG300, HIS304
Cyanidin (1-) CID: 25202542	−19.17	−30.60	-OH (5)	GLU538, GLN550, SER445, ILE446, ALA448, PRO535
Hesperitin-7-methyl ether CID: 14157910	−21.20	−30.90	-OCH_3_(1) -OH (2)	GLU538, PRO535, ALA536, HIS304, ARG300
Naringenin CID: 439246	−20.46	−26.88	-OH (3)	GLU538, ARG300, ALA448, HIS304
Kaempferol 3,5,7-trimethyl ether CID: 14414499	−7.42	−31.61	-OCH_3_(3) -OH (4)	ARG300, ILE446, PRO451, PHE406, ASP447, ALA536, PRO535, ALA448
(+)-Dihydroisorhamnetin CID: 26194552	−21.90	−34.65	-OCH_3_(2) -OH (4)	ARG300, ALA536, VAL537, PHE406, PRO535
Myricetin 3,4′-dimethyl ether CID: 44259718	−14.29	−32.68	-OCH_3_(2) -OH (4)	GLU538, ARG300, ALA448, LEU301, PRO535, PHE406
Quercetin 3,5,5′-trimethyl ether CID: 14162697	−8.30	−37.41	-OCH_3_(2) -OH (3)	ALA448, VAL537, PHE406, PRO535, ALA536, LEU301, ARG300, HIS304
Calycosin CID: 5280448	−19.10	−29.00	-OCH_3_(1) -OH (2)	PRO535, VAL537, PHE406, ALA536, ARG300, LEU301, ALA448
Fomononetin CID: 5280378	−17.50	−27.70	-OCH_3_(1) -OH (1)	HIS403, PHE406, VAL537, ARG300, LEU301, ALA448, HIS304
Genistein CID: 5280961	−18.18	−25.76	-OH (3)	HIS304, GLU538, ALA448, VAL537, PRO535
Chrysin-5-methylether CID: 5490127	−19.38	−30.59	-OH (1)	ALA448, GLU538, PHE406, VAL537, PRO535
Jaceosidin CID: 5379096	−20.66	−30.17	-OCH_3_(2) -OH (3)	VAL537, PHE406, GLU538, HIS304, ARG300, ALA448, PRO535,
Diosmetin CID: 5281612	−20.35	−30.72	-OCH_3_(1) -OH (3)	HIS304, ALA448, ALA536, HIS403, PRO535, VAL537, LEU301, ARG300, PHE406
BAT CID: 5362422	−28.32	−26.08	-OH (1)	GLY372, HIS373, HIS374
Arylsulfonamide (**4b**)	−10.08	−47.77	-OH (3)	GLU538, VAL537, ILE446, PRO535, GLY551, ALA448, ALA515, LYS552

## Data Availability

The raw data supporting the conclusions of this article will be made available by the authors upon request.

## Supplementary Materials

The following supporting information can be downloaded at: https://www.mdpi.com/article/10.3390/molecules31061016/s1, Table S1: Summary of the physiochemical properties of selected sixteen flavonoids; Table S2: Summary of the predicted ADME properties and toxicity of sixteen flavonoids. Table S3: Summary of the CDOCKER energy and CDOCKER interaction energy, and the interacting residues between the 16 flavonoids and the spacer domain of the ADAMTS-5 protein complex. Figure S1: Hydrogen bond occupancy highlighting the residues involved in interaction within the Homoeriodictyol–ADAMTS-5 complex during 100 ns simulation. Figure S2: Ramachandran plot for the OpenFold-generated model, highlighted by the spacer domain residues in yellow dots. Figure S3: RMSD and RMSF analysis plots for the OpenFold model for a short 10 ns molecular dynamics simulation.

## Abbreviations

The following abbreviations are used in this manuscript:

ADAM	A Disintegrin and metalloproteinase
ADAMTS	A Disintegrin and metalloproteinase with thrombospondin motifs
ADAMTS5	A Disintegrin and metalloproteinase with thrombospondin motifs 5
Dis	Disintegrin-like domain
ECM	Extracellular matrix
MD	Molecular dynamics
MM_GBSA	Molecular mechanics/generalized Born surface area
OA	Osteoarthritis
RMSD	Root Mean Square Deviation
RMSF	Root Mean Square Fluctuation
Rg	Radius of Gyration
Sp	Spacer domain
TRS	Thrombospondin type 1 sequence repeat
