# Supplementary material for: Computational Insights into Flavonoids for ADAMTS-5 Exosite Inhibition in Knee Osteoarthritis: Docking, MD Simulations, and Machine Learning-Guided Structure Prediction
Source: Molecules. 2026 Mar 18;31(6):1016. doi: 10.3390/molecules31061016 (PMC13029528; doi:10.3390/molecules31061016)
Supplement: Supplementary file 1 [file molecules-31-01016-s001.zip › molecules-4163096-supplementary.pdf]

Supplementary file :

Table S1: Summary of the physicochemical properties of selected sixteen flavonoids.

Flavonoids Pubchem ID	M.Wt (g/mol)	Num. H-bond acceptor (HBA)	Num. H-bond donor (HBD)	Drug-likeness (Lipinski)	TPSA(A²)	Bioavailability score	PAINS score
4′-Methyl-epigallocatechin CID:176920	320.294	7	5	yes, 0 violation	119.61	0.55	0 alert
CyCyanidin (1-) CID:25202542	285.228	6	3	yes, 0 violation	113.96	0.56	0 alert
Homoeriodictyol CID:73635	302.278	6	3	yes, 0 violation	96.22	0.55	0 alert
Hesperitin-7-methyl ether CID:14157910	316.30534	6	2	yes, 0 violation	85.22	0.55	0 alert
Naringenin CID:439246	272.2527	5	3	yes, 0 violation	86.99	0.55	0 alert
Kaempferol 3,5,7-trimethyl ether CID:14414499	378.31604	6	1	yes, 0 violation	89.42	0.55	0 alert
(+)-Dihydroisorhamnetin CID:26194552	318.278	7	4	yes, 0 violation	116.45	0.55	0 alert
Myricetin 3,4′-dimethyl ether CID:44259718	346.288	8	4	yes, 0 violation	129.59	0.55	0 alert
Quercetin 3,5,3′-trimethyl ether CID:14162697	344.315	7	2	yes, 0 violation	98.36	0.55	0 alert
Biochanin A 7-methylether CID:5386259	298.29	5	1	yes, 0 violation	68.9	0.55	0 alert
Calycosin CID:5280448	284.263	5	2	yes, 0 violation	79.9	0.55	0 alert
Fomononetin CID:5280378	268.264	4	1	yes, 0 violation	59.67	0.55	0 alert

Genistein<br>CID: 5280961	270.236	5	3	yes, 0 violation	90.9	0.55	0 alert
Chrysin-5-methylether<br>CID:5490127	268.264	4	1	yes, 0 violation	59.67	0.55	0 alert
Jaceosidin<br>CID:5379096	330.288	7	3	yes, 0 violation	109.36	0.55	0 alert
Diosmetin<br>CID:5281612	300.2628	6	3	yes, 0 violation	100.13	0.55	0 alert

Table S2: Summary of the predicted ADME properties and toxicity of sixteen flavonoids

Flavonoids Pubchem ID	ADMET_Solubility_level	ADMET_Solubility	ADMET_BBB_Level	Alog P98	ADMET_EXT_CYP2D6	ADMET_EXT_Hapatotoxic	ADMET_EXE_PPB	TOPKAT_Ames_Mutagenicity	TOPKAT_Ames_Probability	TOPKAT_Ames_Score
4′-Methyl-epigallocatechin CID:176920	3	-2.81	4	2.01	0.41	-0.64	-5.51	Non-Mutagen	0.55	-6.43
Cyanidin (1-) CID:25202542	4	-1.27	3	0.10	-7.00	-1.34	-4.93	Non-Mutagen	0.60	-5.16
Homoeriodictyol CID:73635	3	-3.17	3	2.36	1.06	-3.35	-4.65	Non-Mutagen	0.03	-21.37
Hesperitin-7-methyl ether CID:14157910	3	-3.51	3	2.58	0.25	-1.16	-2.34	Non-Mutagen	0.00	-28.05
Naringenin CID:439246	3	-2.94	3	2.37	0.57	-2.21	-5.95	Non-Mutagen	0.02	-23.53
Kaempferol 3,5,7-trimethyl ether CID:14414499	3	-3.73	3	2.55	-1.36	1.40	-2.02	Non-Mutagen	0.70	-1.73
(+)-Dihydroisorhamnetin CID:26194552	3	-2.75	4	1.71	-0.89	-3.35	-7.13	Non-Mutagen	0.41	-9.54

Myricetin 3,4′-dimethyl ether CID:44259718	3	-3.20	4	1.84	-0.51	1.34	-5.52	Non-Mutagen	0.70	-1.74
Quercetin 3,5,3′-trimethyl ether CID:14162697	3	-3.48	3	2.31	-1.19	-1.92	-2.41	Non-Mutagen	0.66	-3.25
Biochanin A 7-methylether CID:5386259	3	-3.62	2	2.59	-0.28	3.84	-0.06	Non-Mutagen	0.08	-18.36
Calycosin CID:5280448	3	-3.18	3	2.37	-1.09	0.44	-3.06	Non-Mutagen	0.05	-19.67
Fomononetin CID:5280378	3	-3.56	2	2.61	-0.36	2.31	-2.01	Non-Mutagen	0.01	-23.98
Genistein CID: 5280961	3	-2.74	3	2.14	0.40	2.40	-4.84	Non-Mutagen	0.07	-18.59
Chrysin-5-methylether CID:5490127	3	-3.81	2	2.88	0.24	-0.48	1.44	Non-Mutagen	0.23	-13.46
Jaceosidin CID:5379096	3	-3.40	3	2.38	-0.62	-1.05	1.26	Non-Mutagen	0.04	-20.84
Diosmetin CID:5281612	3	-3.19	3	2.39	1.13	-0.07	-0.55	Non-Mutagen	0.11	-16.93

Table S3 : Summary of the CDOCKER binding energy and CDOCKER interaction energy, and the interacting residues between the 16 flavonoids and at the Spacer of ADAMTS-5 protein complex.

Flavonoids	CDocker Energy (kcal/mol)	CDocker Interaction energy (kcal/mol)	Interacting Residues
Homoeriodictyol CID: 73635	-14.74	-23.49	SER824, HIS757, GLY820, HIS819, LYS759, ILE758
Biochanin A 7-methyl ether CID: 5386259	-5.45	-19.05	ILE758, ALA755, SER824, THR756, HIS757, GLY822
4′-Methyl-Epigallocatechin CID:176920	-10.29	-26.08	GLY822, MET821, GLY820, THR756, SER824
Cyanidin (1-) CID: 25202542	-14.21	-23.6	GLY820, LYS759, GLY822, THR756, TYR823, HIS757
Hesperitin-7-methyl ether CID: 14157910	-12.52	-22.7	PHE850, GLY820, MET821, VAL805, GLY822, HIS757, SER824
Naringenin CID: 439246	-13.09	-19.79	SER824, HIS757, GLY822
Kaempferol 3,5,7-trimethyl ether CID: 14414499	+1.42	-18.71	SER824, GLY822, HIS757, THR756
(+)-Dihydroisorhamnetin CID: 26194552	-15.21	-26.64	SER824, MET821, HIS819, GLY820, HIS757, GLY822, THR756
Myricetin 3,4′-dimethyl ether CID: 44259718	-5.96	-23.25	HIS819, GLY822, HIS757, SER824, LYS759
Quercetin 3,5,5′-trimethyl ether CID: 14162697	-4.9	-27.14	SER824, MET821, HIS757, LYS759, GLY822
Calycosin CID: 5280448	-6.00	-15.77	TYR823, HIS757, SER824

Fomononetin<br>CID: 5280378	-7.06	-18.98	GLY822, HIS757, ALA755, ILE758, THR756, SER824
Genistein<br>CID: 5280961	-13.076	-21.63	SER824, GLY822, GLY820, HIS757, LYS759,
Chrysin-5-methylether<br>CID: 5490127	-7.57	-18.76	GLY822, LYS759, HIS757
Jaceosidin<br>CID: 5379096	-13.2	-25.63	GLY822, SER824, MET821, LYS759, HIS757
Diosmetin<br>CID: 5281612	-11.75	-19.24	SER824, HIS757, GLY822, TYR823, HIS819,

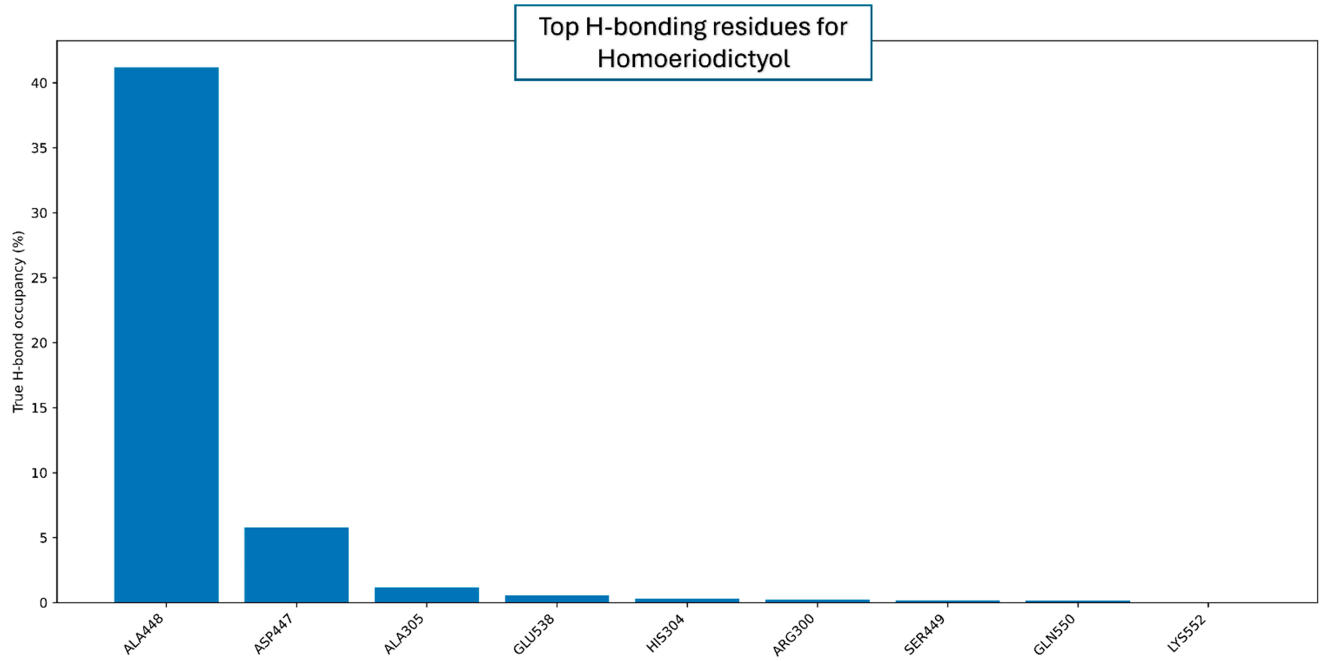

Figure S1 : Hydrogen bond occupancy highlighting the residues involved in interaction for Homoeriodictyol-ADAMTS-5 complex during 100ns simulation.

Ramachandran Plot for OpenFold Model

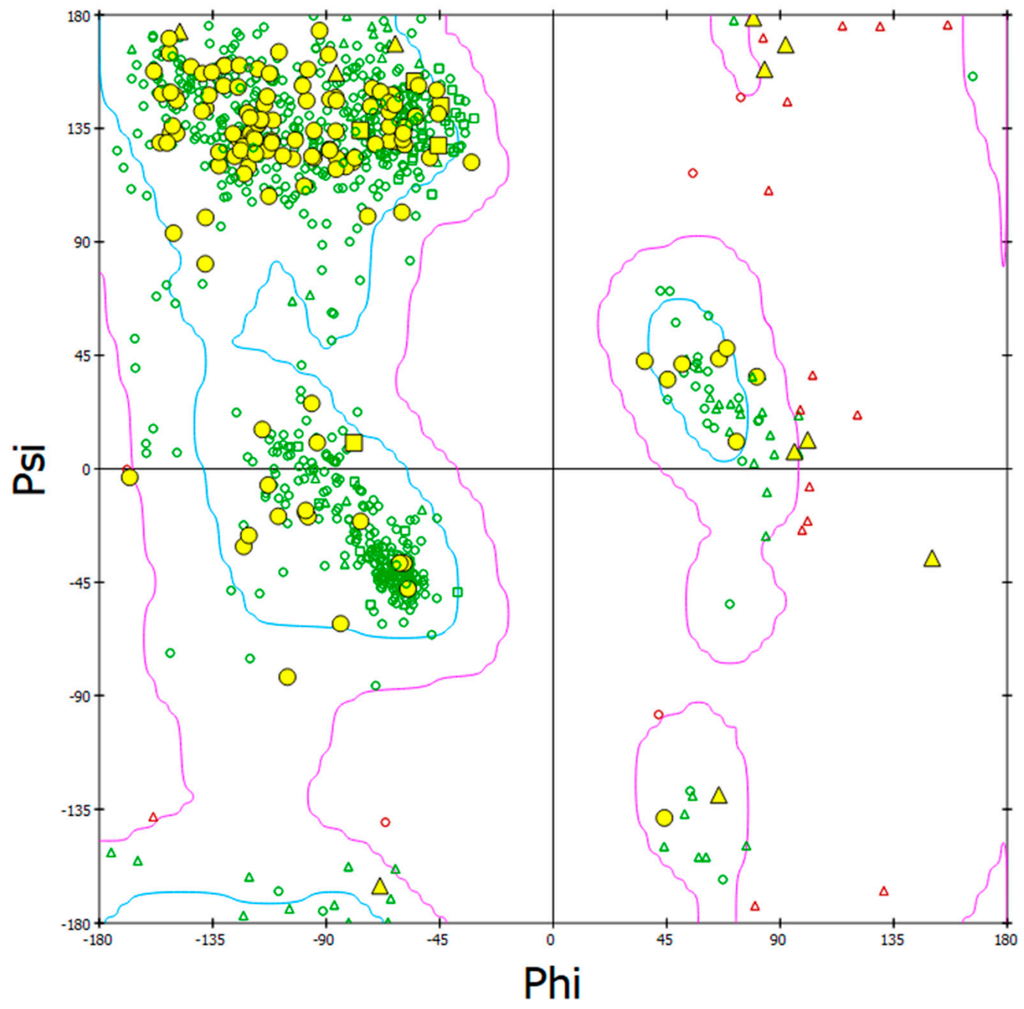

Figure S2 : Ramachandran plot for OpenFold generated model, highlighted by the Spacer Domain residues in yellow dots.

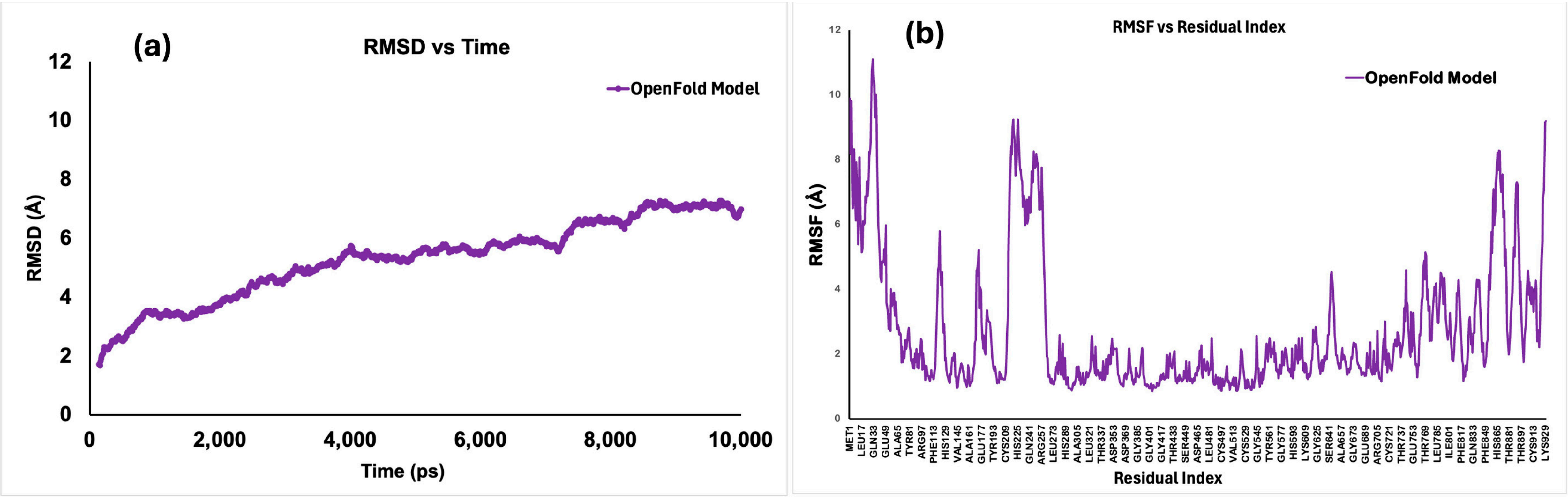

Figure S3 : RMSD and RMSF analysis plots for the OpenFold Model for a short 10ns Molecular Dynamic Simulation.
